# Age at migration and cognitive functioning in later life: a cross-sectional study among older adults in India

**DOI:** 10.1186/s12877-026-07378-x

**Published:** 2026-03-24

**Authors:** Vasim Ahamad, Manoj Dakua, Raza Mohammad

**Affiliations:** 1 Department of Migration and Urban Studies, International Institute for Population Sciences, Govandi Station Road, Deonar, Mumbai, 400088 India; 2 International Institute for Population Sciences, Govandi Station Road, Deonar, Mumbai, 400088 India; 3 Department of Public Health and Mortality Studies, International Institute for Population Sciences, Govandi Station Road, Deonar, Mumbai, 400088 India

**Keywords:** Age at migration, Cognitive functioning, Older adults, LASI, India

## Abstract

**Background:**

Migration can occur at any age and has long-term repercussions on health and well-being later in life. This study aims to assess cognitive functioning ability in later life and its association with age at migration among older adults in India.

**Methods:**

For the analysis, 30,800 respondents aged 60 and above were selected from LASI (Longitudinal Ageing Study in India) data, wave 1. Cognitive functioning ability was measured through five global domains (memory, orientation, arithmetic function, executive function, and object naming). The overall score ranged between 0 and 43, and the higher scores indicate better cognitive functioning. Descriptive statistics, along with mean cognitive scores, were presented by bivariate analysis. Multivariable linear regression models were applied to show the association between age at migration and the cognitive functional abilities of older adults, and results from regression were presented by a beta coefficient (β) with a 95% confidence interval (CI).

**Results:**

Over half of the older persons (55.9%) were growing older as migrants in India. The migrants showed a lower cognition mean score (23) than non-migrants (25). Compared to the non-migrant group, the cognitive score was worse for those who migrated at an age between 0 and 14 years in both the unadjusted (β = -1.93; 95%CI: -2.10 to -1.75) and the adjusted (β = -0.41; 95%CI: -0.56 to -0.26) models. Further, migration during midlife was not found to be significantly associated with cognitive functioning, while migration at age 60 and above shows a significantly lower cognitive score. Similar results were found among male and female cognitive association with age at migration.

**Conclusions:**

The findings of our study reveal that persons with migration status had lower cognitive ability than non-migrants. The age at migration influences cognitive functioning. However, this study predicts that migrants, especially, need attention in cognitive ageing as they have worse cognition than non-migrants.

## Introduction

Cognitive function is a mental capability essential for daily life activities; it includes mental processes such as memory, orientation, verbal fluency, arithmetic abilities, executive function, and object identification [1]. Declines in cognitive functioning are common in older age and may be caused by ageing-related factors and associated physical or psychological disorders [2, 3]. Studies have demonstrated a positive association of cognitive functioning with health and well-being in older age [4, 5]. Furthermore, lower cognitive abilities are recognized as a risk factor, leading to a reduction in social relationships, physical and mental disabilities, functional independence, and even mortality [6, 7]. Age-related cognitive decline is inevitable to some degree. However, various individual and contextual factors significantly influence its rate and extent, including socioeconomic status, health behaviours, and environmental exposures [8]. Factors such as higher educational level, occupational status, higher social support, and urban residence are associated with better cognition in later life [9–11]. Exposure to negative events, such as living in rural residence, hunger, malnutrition, and multimorbidity, might cause an increase in the risk of lower cognitive function at an older age [12, 13]. Migration may influence cognitive function due to changes in socioeconomic resources, environmental factors, and lifestyles [10]. Migration is a process of changing the usual place of residence that can occur at any time during the life course and can have lasting consequences for health and well-being [14, 15].

Migration has emerged as a critical factor in shaping cognitive trajectories in older adults. While migration can offer opportunities for socioeconomic advancement, it often presents challenges that can impair cognitive decline, especially in older age [16]. The age at which individuals migrate has been recognized as a key determinant of health outcomes in later life, particularly cognitive health. Early-life migration may disrupt educational attainment and social integration, which are protective factors against cognitive decline [17]. Conversely, migration in later life often involves significant stressors such as acculturation difficulties, social isolation, and changes in socio-economic status, which can negatively impact cognitive function [18]. The “age at migration” hypothesis posits that the cognitive consequences of migration may vary depending on the life stage at which migration occurs, with critical windows during early and later life being particularly impactful [18, 19]. Migrating late in one’s life has important implications for cognitive health over the life course [16].

Research on migration and cognitive health in low and middle-income countries, including India, remains limited. However, studies in other contexts have consistently shown that migration can have long-term impacts on cognitive ageing. For example, studies of older Mexican Americans have found that those who migrated later in life had higher rates of cognitive impairment compared to earlier migrants or those who remained in their country of origin [18]. Similarly, research on Chinese immigrants in the U.S. has demonstrated that older adults who migrate later in life experience higher levels of cognitive decline, particularly when they lack adequate social support [16]. The immigrants in America were found to have a higher risk of having cognitive disability compared to natives born, and it also varied with their age of migration [19]. Another study found that Mexican American men who immigrated to the United States in middle age had better baseline cognitive function and a slower cognitive decline than U.S.-born Mexican-Americans [20]. Moreover, studies from various countries show that residential mobility in the life course is also associated with cognitive functioning in later life. Recent studies in China have found that residential changes due to migration are associated with cognitive status; urban-to-urban residents had higher cognitive levels than rural-to-rural migrants [21]. Similar results were also found among studies conducted in India [9, 11]. However, regional-level studies show that within-country migration influences later-life cognitive function in India [22–24]. However, studies on migration and cognitive functioning at the national level in India remain limited. Building on this gap, the present study investigates how the timing of migration, specifically, age at migration, shapes cognitive health in later life. Using nationally representative data, this analysis offers new insights into how life-course migration histories influence ageing outcomes in the Indian context.

In developing countries such as India, migration patterns are complex and largely driven by economic necessity. Internal migration, particularly rural-to-urban movement, is prevalent and often occurs in poor living conditions, inadequate social support, and limited access to healthcare, all of which can contribute to poorer cognitive outcomes [25, 26]. Older adults in these contexts may be particularly vulnerable, as they face compounded risks of economic marginalization, social isolation, and health disparities [27]. The ageing in India is experiencing a demographic transition; 103 million people are 60 and above age, which increased from 5.6% in 1961 to 8.6% in 2011, of the total population of India [28]. With the increase in the older population, the share of older migrants also increased in India, and 53 million people aged 60 + were migrants, which is 51% of the total older population. From the 2001 census to 2011, the number of older migrants changed from 34.6 million to 53.8 million [29].

More than half of the older persons in India were migrants, but how migration and migration-related factors influence cognitive function among older people has not yet been explored. Recent research from national-level cross-sectional data has shown significant gender and geographic differences in late-life cognitive function among older Indian adults [24, 30]. However, the differences in cognition were not fully explained by gender and migration trajectories as life course perspectives. Given India’s unique demographic and migration patterns, understanding the specific mechanisms through which migration influences cognitive outcomes in this context is crucial. This study seeks to address this gap by investigating the relationship between age at migration and cognitive functioning among older adults in India. We anticipated that, compared to non-migrants, older adults in India who migrated, particularly during early or late life, may exhibit worse cognitive scores, based on life-course and cumulative disadvantage frameworks, while acknowledging that mid-life migration could have mixed effects depending on socioeconomic and health factors. By examining the cognitive outcomes of migrants who migrated at different life stages, this research aims to inform public health interventions to promote cognitive health among India’s ageing population.

## Method and materials

### Data source

**Fig. 1:**
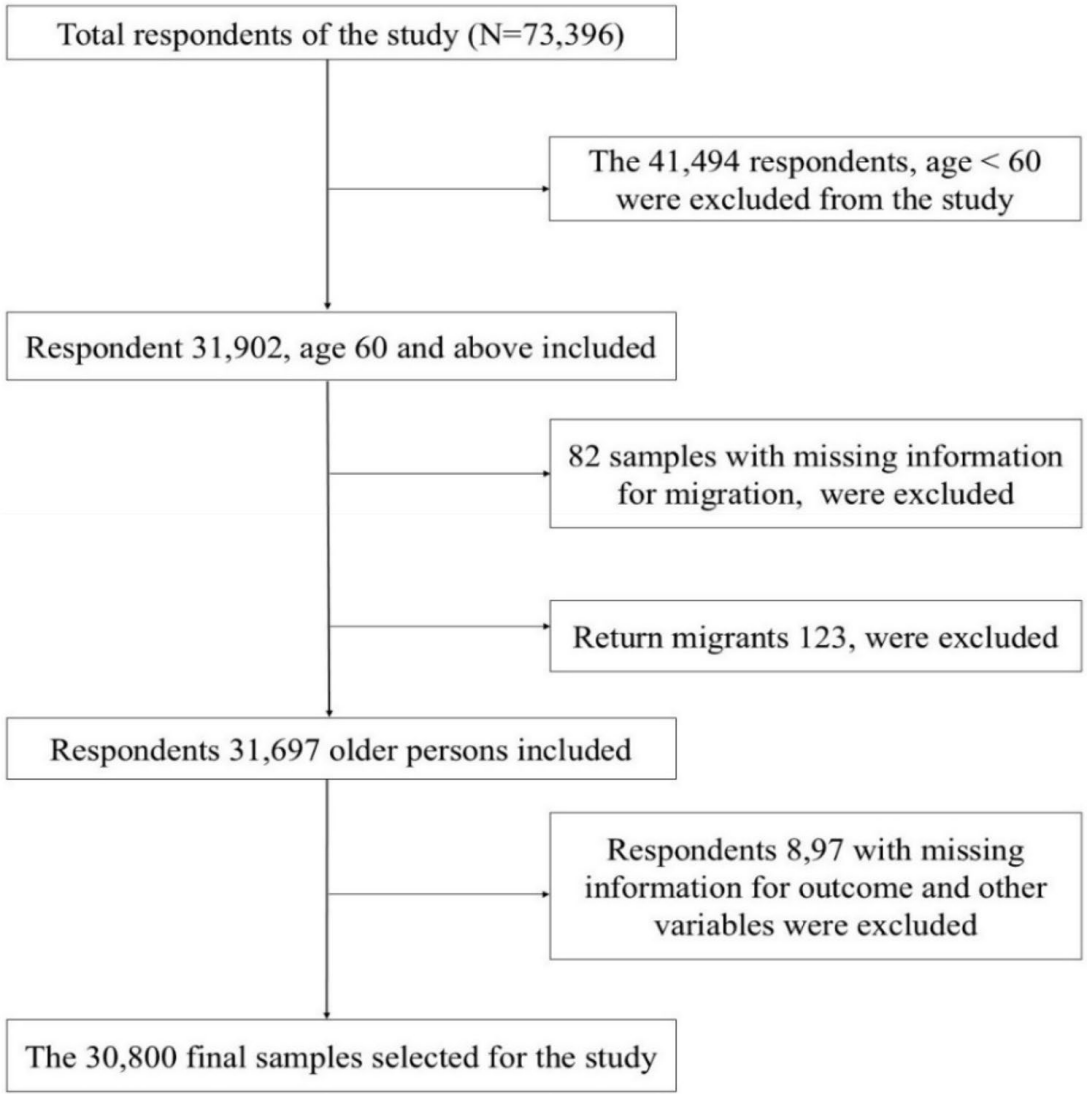
Sample selection for the study, LASI-I (2017-18)

A cross-sectional study design was adopted for this study. Data for the analysis were drawn from the Longitudinal Ageing Study in India (LASI), wave-1, collected from 2017 to 2018. It is a nationally representative survey of 73,396 individuals, 31,135 males and 42,261 females aged 45 years and above, and their spouses (regardless of age) across all states and union territories of India [31]. This survey has been conducted to study the health status and socioeconomic well-being of older adults in India. A multistage stratified area probability cluster sampling design was applied to the LASI survey to determine the eventual observation units. Within each state, LASI Wave-1 adopted a three-stage sampling design in rural areas and a four-stage sampling design in urban areas. In each state/UT, the first stage involved the selection of Primary Sampling Units (PSUs), that is, sub-districts (Tehsils/Talukas), and the second stage involved the selection of villages in rural areas and wards in urban areas in the selected PSUs. In rural areas, households were selected from selected villages in the third stage. However, sampling in urban areas involved an additional stage. Specifically, in the third stage, one Census Enumeration Block (CEB) was randomly selected in each urban area. In the fourth stage, households were selected from this CEB. In the LASI individual questionnaire, the questions related to migration were asked as follows: (1) How many years have you been living (continuously) in this area (2)? Where is your place of birth (3)? Where were you living before coming to this place (place of the last residence)? The three questions here help to identify the migrant and non-migrant groups. If the place of birth (POB) and last residence (POLR) differed from the place of enumeration at survey time, then the person is considered a migrant. In the present study, we classified migrants based on their place of last residence (POLR) (Where were you living before coming to this place), as this question also encompasses multiple and return migrations, and return migrants were excluded from this study. This study includes both internal and international migrants. The 30,800 respondents were selected for the study; the inclusion and exclusion criteria for the respondents are shown in Fig. 1.

Migration refers to the temporary or permanent movement of individuals from their usual place of residence to another location. Individuals who change their usual place of residence are referred to as migrants. In the context of ageing research, older migrants are defined as individuals aged 60 years and above who have experienced migration at any stage of their life course and are residing at a destination location in later life.

### Outcome variables

Older persons’ cognitive functional status is the study’s primary outcome variable. In the LASI survey, the five domains, such as memory, orientation, arithmetic function, executive function, and objective naming, were selected to assess the cognitive functional health of middle and older adults. Measuring these five domains and their score is discussed as follows.

1. Memory: Immediate word recall (score 0–10) and delayed word recall (score 0–10) were used to assess memory cognition. The total memory score ranged from 0 to 20.
2. Orientation: The orientation was measured using the time (score 0–4) and place (score 0–4). The overall score for orientation ranged from 0 to 8.
3. Arithmetic function: Backward counting (score 0–2), serial 7 (score 0–5), and computation were used to evaluate the arithmetic function (score 0–2). The arithmetic function’s overall score ranged from 0 to 9.
4. Executive function: Paper folding (scoring 0–3) and pentagon drawing (score 0–1) were used to assess executive function. The overall score for executive function ranged from 0 to 4.
5. Object naming: The scores for object naming ranged from 0 to 2.

The cognitive index was constructed by combining all five dimensions of scores. The overall score of the composite cognitive index varied from 0 to 43 (mean 24), with a higher mean showing better cognitive ability and vice versa. Further information on the cognitive function assessment can be seen from the LASI 2020 report [31].

### Key explanatory variables

The primary explanatory variable of interest in the study is age at migration. The migration of older persons is defined based on the question of the place of last residence (POLR). According to the Indian census definition, a person is considered a migrant if his/her Place of Last Residence (POLR) differs from the place of enumeration (POE) where he/she is being enumerated [32]. Accordingly, this study follows the standard Census of India definition and classifies individuals as migrants if they have moved from their usual place of residence, village or town, to another place.

#### Age at migration

The age at migration is defined as the age at which a person migrates from one place to another; it can be a vital and valuable aspect for understanding the person’s health disparities in middle and old age. For this study, we measure age at migration based on a person’s duration of migration and their current age. The age at migration is categorized into four groups: migration occurs at ages 0–14 (early age), 15–44 (young and middle age), 45–59 (pre-retirement age), and 60 and above (older age) among older persons.

### Other explanatory variables

Potential confounders of cognitive function, such as socioeconomic and health characteristics at both individual and household levels, are included in the analysis to adjust results based on previous studies ([9, 11, 33]. The categorization of covariates is discussed as follows.

Age (60–69, 70–79, and 80+); Sex (male and female); Place of residence (rural and urban); Marital status (currently married, widowhood, and others); Education (No education, primary, secondary and higher secondary, and graduate and above); Religion (Hindu, Muslim, and others); Currently working (working, and not worked); Wealth status (poor, middle, rich); Living arrangement (living with children and spouse, living with others, and living alone); Smoking (no, yes); Alcohol (no, yes); Physical activity (no, yes); Self-rated health (good, poor); Functional ability (good, moderate, poor).

Wealth status are measures based on the MPCE (monthly per capita expenditure) quintile. Self-reported health shows the overall health of persons and is categorized in this study as “good” and “poor”. Functional limitation was defined as difficulty in daily living (ADL) and instrumental activity difficulty (IADL). A total of twelve questions for difficulties (difficulties in Dressing, Walking, Bathing, Eating, In and Out of bed, Using the toilet, Preparing Meals, Shopping for groceries, making telephone calls, taking medications, doing work, and managing money) were included to generate physical disability (reliability coefficient or Cronbach’s alpha = 0.88). They were recoded into yes and no (“yes” as 1 = having difficulties, and “no” as 0 = no difficulties). A total 0–12 score categorized the functional ability as good “0 difficulties”, moderate “1–3 difficulties”, and poor functional ability “4–12 difficulties”.

### Statistical analysis

The study used descriptive statistics and bivariate analysis to examine the characteristics of the variables with migration status. The t-test was used to compare the mean cognitive score among different categories of covariates. Aside from that, the findings of the association of older persons’ cognitive score with age at migration and selected independent variables were carved out using a multivariable linear regression model. A linear regression model can be written as follows:

$$\begin{aligned}\:Y\:\left(Cognitive\:score\right)&=\:{\beta\:}_{0}+{\beta\:}_{1}Age\:at\:migration+{\beta\:}_{2}Age\\ &+{\beta\:}_{3}Sex+{\beta\:}_{4}Residence+\\ &{\beta\:}_{5}Marital\:status+{\beta\:}_{6}Education+\\ &{\beta\:}_{7}Caste+{\beta\:}_{8}Religion+{\beta\:}_{9}Living\:arrangement+\\ &{\beta\:}_{10}Wroking\:status+{\beta\:}_{11}Wealth\:status+\\ &{\beta\:}_{12}Smoking+{\beta\:}_{13}Alcohol+{\beta\:}_{14}Physical\:activity+\\ &{\beta\:}_{15}Functinal\:ability+{\beta\:}_{16}Self-rated\:health\end{aligned}$$

Where, Age at migration, Age, Sex, Residence, Marital status, Education, Caste, Religion, Living arrangement, Working status, Wealth status, Smoking, Alcohol, Physical activity, Functional ability, and Overall health the set of explanatory variables, and β_1,_ β_2,_ β_3, −−−−−−−−_ β_16_ are the regression coefficients to be estimated in the model [34].

The regression analysis estimates were presented as coefficients with a 95% confidence interval. The proper individual-level sampling weights were used to make the results representative. The statistical package STATA for Windows version 16 was used for all statistical analyses. All the variables were tested for multicollinearity before being included in the regression analysis, and we found no multicollinearity among the variables. We also checked the data’s linearity and found that the data was normally distributed. All linear regression models were estimated using robust standard errors to address potential heteroscedasticity, ensuring valid statistical inference. As a sensitivity check, standard errors were also clustered at the state level to account for the hierarchical nature of the LASI data (individuals nested within states). The clustering did not materially change the results, confirming the robustness of the findings.

## Results

### Background characteristics of older adults

Table 1 depicts the descriptive characteristics of 30,800 older adults in India, consisting of 17,352 migrants and 13,448 non-migrants. The sample was predominantly aged 60–69 years with an equal distribution by sex. Most participants resided in rural areas, though migrants were less likely to do so than non-migrants. The majority were currently married, had no formal education, belonged to other backward classes, and identified as Hindu. Living with family and not working were common. A substantial proportion came from poor households, reported no history of smoking or alcohol use, low physical activity, and about half rated their overall and functional health as good. Among older migrant adults, more than 80% had migrated between 0 and 14 years, and over 60% had moved from one rural area to another.

**Table 1:** Background characteristics of the older adults in India, LASI wave-1 (2017-18)

Variables	Migrants		Non-migrants		Total		Chi-square result		
	Number	%	Number	%	Number	%	χ²	df	Cramer’s V
Age**									
60–69	10,512	60.6	7,785	57.9	18,297	59.4	8.64	2	0.02
70–79	5,119	29.5	4,078	30.3	9,198	29.9			
80+	1,721	9.9	1,585	11.8	3,305	10.7			
Sex***									
Male	4,314	24.9	10,234	76.1	14,548	47.2	5200.00	1	-0.41
Female	13,038	75.1	3,214	23.9	16,252	52.8			
Residence***									
Rural	11,381	65.6	10,480	77.9	21,860	71	733.56	1	-0.15
Urban	5,971	34.4	2,968	22.1	8,940	29			
Marital status***									
Currently married	9,597	55.3	9,455	70.3	19,051	61.9	586.51	2	0.08
Widowed	7,467	43	3,633	27	11,099	36			
Others	289	1.7	361	2.7	649	2.1			
Education***									
No-education	10,834	62.4	6,602	49.1	17,435	56.6	176.15	3	0.08
Primary	3,487	20.1	3,529	26.2	7,016	22.8			
Secondary and higher secondary	2,386	13.8	2,702	20.1	5,088	16.5			
Graduate and above	645	3.7	616	4.6	1,261	4.1			
Caste category									
Scheduled caste	3,397	19.6	2,477	18.4	5,874	19.1	322.63	3	0.10
Scheduled tribes	1,244	7.2	1,242	9.2	2,486	8.1			
Other backward class	7,649	44.1	6,307	46.9	13,956	45.3			
Others	5,063	29.2	3,421	25.4	8,484	27.6			
Religion***									
Hindu	14,389	82.9	11,091	82.5	25,480	82.7	67.30	2	0.05
Muslim	1,705	9.8	1,589	11.8	3,294	10.7			
Others	1,259	7.3	768	5.7	2,027	6.6			
Living arrangement***									
Living with children and spouse	15,190	87.5	12,185	90.6	27,375	88.9	25.28	2	0.03
Living with others	1,012	5.8	652	4.9	1,664	5.4			
Living alone	1,149	6.6	611	4.6	1,760	5.7			
Working status***									
Currently not working	13,164	75.9	7,965	59.2	21,129	68.6	957.19	1	-0.18
Currently working	4,188	24.1	5,483	40.8	9,671	31.4			
Wealth status***									
Poor	7,368	42.5	6,031	44.9	13,399	43.5	19.56	2	0.03
Middle	3,659	21.1	2,724	20.3	6,383	20.7			
Rich	6,325	36.5	4,693	34.9	11,018	35.8			
Health Condition									
Ever smoked***									
No	12,091	69.7	6,314	47	18,405	59.8	1100.00	1	0.19
Yes	5,261	30.3	7,134	53.1	12,395	40.2			
Ever alcohol***									
No	15,877	91.5	10,419	77.5	26,296	85.4	973.71	1	0.18
Yes	1,475	8.5	3,029	22.5	4,504	14.6			
Physical activity***									
No	13,576	78.2	9,038	67.2	22,613	73.4	471.55	1	0.12
Yes	3,776	21.8	4,410	32.8	8,187	26.6			
Self-rated health***									
Good	8,597	49.5	7,231	53.8	15,828	51.4	82.80	1	-0.05
Poor	8,755	50.5	6,217	46.2	14,972	48.6			
Functional ability***									
Good	8,068	46.5	7,409	55.1	15,477	50.3	243.86	2	0.09
Moderate	3,764	21.7	2,775	20.6	6,540	21.2			
Poor	5,519	31.8	3,264	24.3	8,783	28.5			
Age at migration									
0–14	13,903	81			13,903	81			
15–44	781	4.6			781	4.6			
45–59	1,495	8.7			1,495	8.7			
60+	990	5.8			990	5.8			
Migration stream									
Rural-Rural	10,590	63.1			10,590	63.1			
Rural-Urban	3,602	21.5			3,602	21.5			
Urban-Rural	442	2.6			442	2.6			
Urban-Urban	2,157	12.8			2,157	12.8			
Total	30,800	100	17,352	100	13,448	100			

χ² = Chi-square test statistic; df = degrees of freedom; Cramer’s V indicates effect size test, LASI Individual sample weights were applied

Note: *** *p* = < 0.001, **p = < 0.05, & * *p* = < 0.1, *P*-values are based on the Chi-square (χ²)

## Cognitive functional ability of the older adults

Table 2 shows the mean cognitive scores of migrant and non-migrant older adults in India, revealing significant differences between the two groups across various demographics. Cognitive scores generally declined with age, but notable differences were observed between migrants and non-migrants in each age group. Among older adults, migrants generally exhibited lower mean cognitive scores than non-migrants across most socio-demographic and health characteristics. Although male migrants had slightly higher scores than non-migrant males (27.39 vs. 26.28), migrants living in rural areas (21.65), those who were widowed (21.17), poor (22.06), or had no formal education (19.90) showed notably lower cognitive performance. Migrants who were married (25.30), working (24.67), or living with children and spouses (23.95) also scored lower than their non-migrant counterparts. Importantly, even among relatively healthier groups, such as non-smokers, non-drinkers, physically active individuals, and those reporting good functional or overall health, migrants consistently had lower mean cognitive scores than non-migrants.

**Table 2:** Cognitive functional ability status of migrants and non-migrant older adults with background characteristics in India, LASI wave-1 (2017-18)

Variables	Migrants mean	Non-migrants mean	mean difference	Total mean	t-test result			
					*p*-value	t	df	Cohen’s d
Total	23.67	25.01	-1.34	24.26	< 0.001	-15.58	30,798	0.18
Age								
60–69	24.90	26.33	-1.43	25.52	< 0.001	-13.73	18,759	0.20
70–79	22.65	24.02	-1.37	23.27	< 0.001	-8.58	8887	0.18
80+	18.95	20.28	-1.33	19.56	< 0.001	-4.74	3148	0.17
Sex								
Male	27.39	26.28	1.11	26.66	< 0.001	9.08	14,803	0.16
Female	22.09	21.88	0.20	22.04	0.126	1.53	15,993	0.03
Residence								
Rural	21.65	24.08	-2.43	22.86	< 0.001	-24.22	20,384	0.34
Urban	26.64	27.71	-1.06	27.00	< 0.001	-6.98	10,412	0.14
Marital status								
Currently married	25.30	26.15	-0.86	25.72	< 0.001	-8.37	19,655	0.12
Widowed	21.17	22.07	-0.89	21.48	< 0.001	-5.86	10,341	0.12
Others	24.98	23.94	1.04	24.44	0.0705	1.81	798	0.13
Education								
No-education	19.92	21.06	-1.13	20.39	< 0.001	-11.34	16,547	0.18
Primary	26.03	26.87	-0.84	26.43	< 0.001	-5.89	7420	0.14
Secondary and higher secondary	30.78	30.75	0.02	30.77	0.8657	0.17	5412	0.00
Graduate and above	33.29	33.24	0.05	33.27	0.8354	0.21	1413	0.01
Caste category								
Scheduled caste	22.05	24.25	-2.20	22.98	< 0.001	-11.26	4993	0.32
Scheduled tribes	20.82	22.13	-1.31	21.53	< 0.001	-6.09	5144	0.17
Other backward class	23.82	25.48	-1.66	24.56	< 0.001	-12.14	11,773	0.23
Others	25.57	27.10	-1.53	26.16	< 0.001	-9.56	8882	0.21
Religion								
Hindu	23.68	25.36	-1.68	24.40	< 0.001	-16.67	22,516	0.22
Muslim	23.14	25.04	-1.90	24.06	< 0.001	-7.78	3582	0.26
Others	23.98	23.49	0.50	23.75	0.0295	2.18	4696	0.06
Living arrangement								
Living with children and spouse	23.95	25.26	-1.31	24.54	< 0.001	-14.53	27,628	0.18
Living with others	20.91	22.90	-1.99	21.74	< 0.001	-5.11	1592	0.26
Living alone	21.82	22.22	-0.41	21.98	0.3014	-1.03	1574	0.05
Working status								
Currently working	24.67	26.04	-1.37	25.46	< 0.001	-9.34	9194	0.20
Currently not working	23.37	24.35	-0.98	23.75	< 0.001	-9.11	21,602	0.13
Wealth status								
Poor	22.06	23.99	-1.93	22.95	< 0.001	-14.70	12,678	0.26
Middle	23.76	25.09	-1.32	24.34	< 0.001	-7.01	6284	0.18
Rich	25.25	26.13	-0.88	25.63	< 0.001	-6.32	11,832	0.12
Health Condition								
Ever smoked								
No	23.83	24.89	-1.06	24.22	< 0.001	-9.11	18,873	0.14
Yes	23.30	25.14	-1.83	24.33	< 0.001	-13.90	11,923	0.26
Ever alcohol								
No	23.51	24.98	-1.46	24.10	< 0.001	-15.20	25,512	0.19
Yes	24.87	25.11	-0.24	25.02	0.2583	-1.13	5284	0.03
Physical activity								
No	23.25	24.42	-1.17	23.73	< 0.001	-11.40	22,652	0.15
Yes	25.16	26.22	-1.06	25.74	< 0.001	-6.65	8144	0.15
Functional ability								
Good	25.55	26.55	-1.01	26.03	< 0.001	-9.02	16,802	0.14
Moderate	23.47	24.41	-0.94	23.87	< 0.001	-5.30	6285	0.14
Poor	20.39	21.26	-0.86	20.72	< 0.001	-4.97	7707	0.12
Self-rated health								
Good	24.54	25.80	-1.26	25.13	< 0.001	-10.82	16,589	0.17
Poor	22.74	23.97	-1.24	23.25	< 0.001	-9.76	14,207	0.18

Note: *p*-value determined by t-test to see the significance of the mean difference; *t* values are based on t-tests; df = degrees of freedom; Cohen’s d indicates effect size

**Fig. 2:**
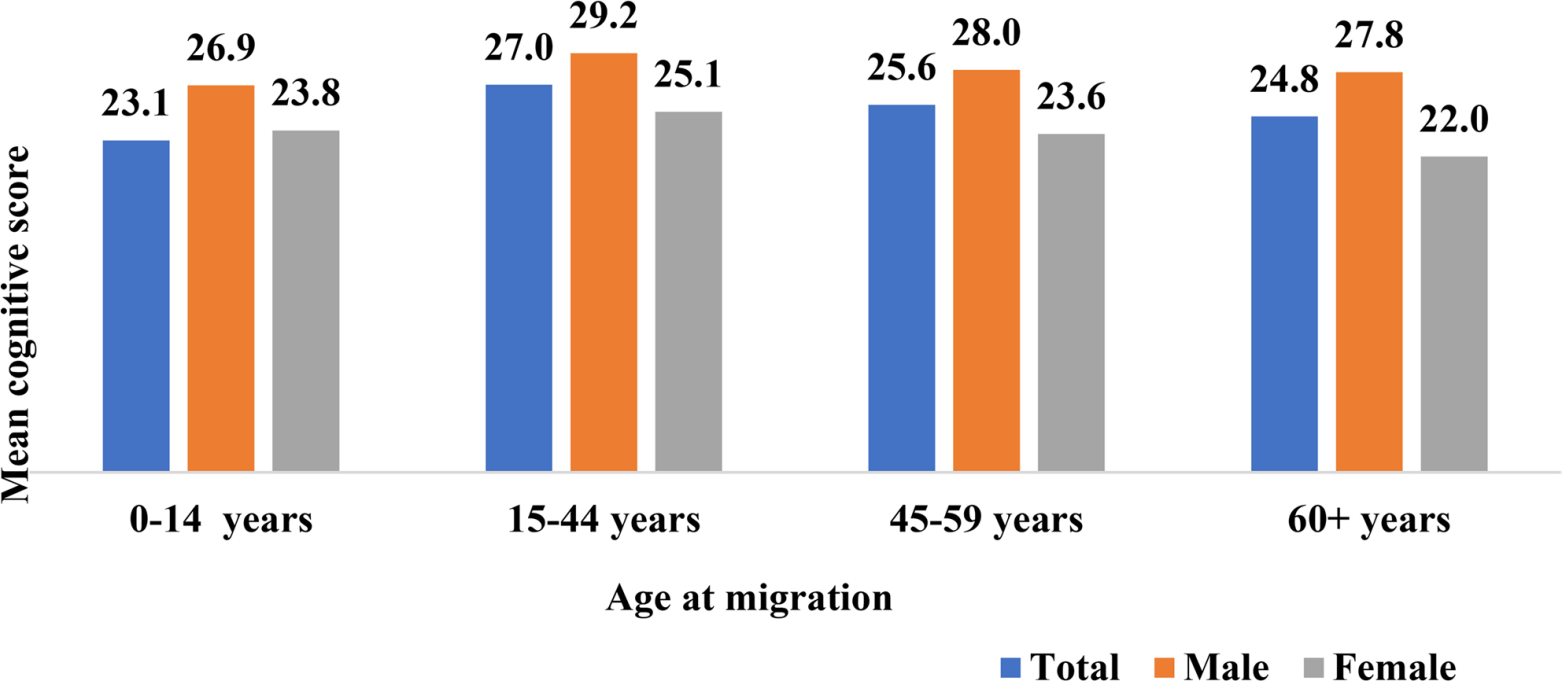
Cognitive status among older adults with age at migration by sex, LASI wave-1 (2017-18)

Figure 2 illustrates the mean cognitive scores of older adults in India based on their age at the time of migration by sex. The analysis shows that older adults who migrated at a very young age (0–14 years) (mean: 23.1) or 60 years (mean: 24.8) and above had lower mean cognitive scores than those who migrated between the ages of 15–44 (mean: 27) and 45–59 (mean: 25.6). This suggests that the age at which migration occurs may influence cognitive outcomes later in life. Further, the figure shows that among males, the cognitive score is higher compared to females of all ages of migration categories, but the mean score varies with age at migration for both males and females, respectively.

### Association of cognitive functional ability with the age at migration

Table 3 depicts the estimated β coefficients from the linear regression analysis of cognitive scores among older adults in India. Three separate models were used to estimate the β coefficients. In Model 1, only the unadjusted effect of migration was considered. Model 2 was adjusted for socio-economic and demographic characteristics, while Model 3 included adjustments for all socio-economic, demographic, and health-related characteristics. The analysis indicated that the unadjusted β coefficients were − 1.93 (β: -1.93; 95%CI: -2.10,-1.75; P: <0.001) and − 0.25 (β: -0.25; 95%CI: -0.71,0.20; P: <0.001) for migrants whose migration occurred at ages 0–14 and 60 or above, respectively, compared to non-migrant older adults. This means that the mean cognitive scores for those who migrated at ages 0–14 and 60 and above were 1.93 and 0.25 points lower than those of non-migrant older adults. When socio-economic and demographic characteristics were considered, the β coefficients were − 0.50 (β: -0.50; 95%CI:-0.65,-0.35; P: <0.001), -0.53 (β: -0.53; 95%CI: -0.90,-0.16; P: <0.001), -0.66 (β: -0.66; 95%CI:-0.96,-0.36; P: <0.001), and − 0.38 (β: -0.38; 95%CI: -0.73,-0.02; P: <0.001) for those who migrated at ages 0–14, 15–44, 45–59, and 60 and above, respectively, compared to non-migrant older adults. This indicates that even after accounting for socio-economic and demographic factors, the mean cognitive scores of migrants were still lower than those of non-migrant older adults across all migration age groups. Furthermore, after adjusting for all socio-economic, demographic, and health characteristics, the mean cognitive scores remained significantly lower for migrants compared to non-migrant older adults: 0.41 points (β: -0.41; 95%CI: -0.56,-0.26; P: <0.001) lower for those who migrated at ages 0–14, 0.46 points (β: -0.46; 95%CI: -0.82,-0.09; P: <0.001) lower for those who migrated at ages 15–44, 0.58 points (β: -0.58; 95%CI: -0.87,-0.29; P: <0.001) lower for those who migrated at ages 45–59 compared to non-migrant older adults. In contrast, although cognitive scores were 0.27 points lower among those who migrated at ages 60 and above (β = −0.27; 95% CI: −0.62, − 0.08), this association was not statistically significant.

**Table 3:** Estimated coefficient for cognitive functional ability to show the association of age at migration among older adults in India, LASI wave-1 (2017-18)

Cognitive status	Model 1		Model 2		Model 3	
	β Coef.	95% CI	β Coef.	95% CI	β Coef.	95% CI
Age at migration						
Non-migrants^*®*^	0	[0.00,0.00]	0	[0.00,0.00]	0	[0.00,0.00]
0–14 years	-1.93***	[-2.10,-1.75]	-0.50***	[-0.65,-0.35]	-0.41***	[-0.56,-0.26]
15–44 years	1.96***	[1.49,2.44]	-0.53**	[-0.90,-0.16]	-0.46*	[-0.82,-0.09]
45–59 years	0.57**	[0.19,0.95]	-0.66***	[-0.96,-0.36]	-0.58***	[-0.87,-0.29]
60 + years	-0.25	[-0.71,0.20]	-0.38*	[-0.73,-0.02]	-0.27	[-0.62,0.08]
Age						
60–69^*®*^			0	[0.00,0.00]	0	[0.00,0.00]
70–79			-1.58***	[-1.73,-1.43]	-1.31***	[-1.46,-1.16]
80+			-4.13***	[-4.36,-3.90]	-3.49***	[-3.72,-3.26]
Sex						
Male^*®*^			0	[0.00,0.00]	0	[0.00,0.00]
Female			-1.44***	[-1.61,-1.28]	-1.42***	[-1.60,-1.25]
Residence						
Rural^*®*^			0	[0.00,0.00]	0	[0.00,0.00]
Urban			1.72***	[1.57,1.87]	1.62***	[1.47,1.77]
Marital status						
Currently married^*®*^			0	[0.00,0.00]	0	[0.00,0.00]
Widowed			-0.94***	[-1.10,-0.78]	-0.78***	[-0.94,-0.61]
Others			-0.56*	[-0.99,-0.12]	-0.43	[-0.86,0.00]
Education						
No-education^*®*^			0	[0.00,0.00]	0	[0.00,0.00]
Primary			4.87***	[4.71,5.04]	4.72***	[4.56,4.89]
Secondary and higher secondary			8.19***	[7.99,8.39]	7.90***	[7.71,8.10]
Graduate and above			10.12***	[9.78,10.46]	9.66***	[9.33,10.00]
Caste category						
Scheduled Caste^*®*^			0	[0.00,0.00]	0	[0.00,0.00]
Scheduled Tribes			-1.90***	[-2.13,-1.66]	-2.07***	[-2.30,-1.84]
Other Backwards Caste			0.37***	[0.17,0.56]	0.33***	[0.14,0.52]
Others			0.33**	[0.12,0.53]	0.30**	[0.10,0.51]
Religion						
Hindu^*®*^			0	[0.00,0.00]	0	[0.00,0.00]
Muslim			0.17	[-0.04,0.37]	0.22*	[0.01,0.42]
Others			0.28**	[0.08,0.48]	0.15	[-0.05,0.34]
Living arrangement						
Living with children and ^*®*^spouse			0	[0.00,0.00]	0	[0.00,0.00]
Living with others			-0.45**	[-0.77,-0.14]	-0.41*	[-0.72,-0.10]
Living alone			0.06	[-0.24,0.37]	0	[-0.30,0.30]
Working status						
Currently working ^*®*^			0	[0.00,0.00]	0	[0.00,0.00]
Currently not working			0.86***	[0.70,1.01]	0.27**	[0.10,0.43]
Wealth status						
Poor^*®*^			0	[0.00,0.00]	0	[0.00,0.00]
Middle			0.60***	[0.43,0.77]	0.56***	[0.39,0.73]
Rich			0.96***	[0.81,1.11]	0.96***	[0.82,1.11]
Health Condition						
Ever smoked						
No^*®*^					0	[0.00,0.00]
Yes					0.03	[-0.12,0.17]
Ever alcohol						
No^*®*^					0	[0.00,0.00]
Yes					-0.42***	[-0.61,-0.23]
Physical activity						
No^*®*^					0	[0.00,0.00]
Yes					0.86***	[0.70,1.02]
Functional ability						
Good^*®*^					0	[0.00,0.00]
Moderate					-0.58***	[-0.75,-0.42]
Poor					-2.01***	[-2.18,-1.85]
Self-rated health						
Good^*®*^					0	[0.00,0.00]
Poor					-0.57***	[-0.71,-0.44]
Constant	25.01***	[24.88,25.13]	22.13***	[21.90,22.36]	22.98***	[22.72,23.24]

Note: *** p = < 0.001, **p = < 0.05, & * p = < 0.1; ^®^= Reference, β coef. = β Coefficient, CI= Confidence Interval

Table 4 shows the estimated β coefficients from the adjusted linear regression analysis of cognitive scores among older adults by sex in India. Two models were used to estimate the β coefficients. The male-specific adjusted model considered the effect of age at migration on cognitive score among older male adults. Female-specific adjusted model represents the effect of age at migration on cognitive score among female older adults. The analysis of Male-specific adjusted model indicated that the adjusted β coefficients for the age at migration were as follows: 0–14 years was − 0.37 (β: -0.37; 95%CI:-0.59,-0.14; P: <0.005), and 45–59 years was − 0.69 (β: -0.69; 95%CI: -1.11,-0.27; P: <0.005). This means that male older adults who migrated at ages 0–14 and 45–59 years had a mean cognitive score of 0.37 and 0.69 points, respectively, lower than that of non-migrant male older adults. Furthermore, The analysis of Female-specific adjusted model indicated that the adjusted β coefficients for the age at migration were as follows: 0–14 years was − 0.42 (β: -0.42; 95%CI:-0.63,-0.20; P: <0.005), 45–59 was − 0.47 (β: -0.47; 95%CI: -0.88,-0.06; P: <0.005) and 60 + years was − 0.61 (β: -0.61; 95%CI: -1.10,-0.12; P: <0.005). This means that female older adults who migrated at ages 0–14, 45–59, and 60 + years had a mean cognitive score of 0.42, 0.47, and 0.61 points, respectively, lower than non-migrant female older adults. The 15–44 years age of migration did not show a significant association for a lower cognitive score in both models for males and females.

**Table 4:** Estimated coefficient for cognitive functional ability to show the association of age at migration among older adults by sex in India, LASI wave-1 (2017-18)

β				
Cognitive status	Male-specific adjusted model		Female-specific adjusted model	
	β Coef.	95% CI	β Coef.	95% CI
Age at migration				
Non-migrants^*®*^	0	[0.00,0.00]	0	[0.00,0.00]
0–14 years	-0.37**	[-0.59,-0.14]	-0.42***	[-0.63,-0.20]
15–44 years	-0.47	[-0.99,0.06]	-0.47	[-0.98,0.03]
45–59 years	-0.69**	[-1.11,-0.27]	-0.47*	[-0.88,-0.06]
60 + years	0.15	[-0.35,0.64]	-0.61*	[-1.10,-0.12]
Constant	23.33***	[22.97,23.69]	21.81***	[21.41,22.20]

Model 1 and Model 2 adjusted by age, residence, marital status, education, caste, religion, living arrangement, working status, smoking, alcohol, physical activity, Functional status, and Self-rated health

Note: *** *p* = < 0.001, **p = < 0.05, & * *p* = < 0.1; ^®^= Reference, β coef. = β Coefficient, CI= Confidence Interval

## Discussion

Cognitive function concerning the age at migration of older persons is one of the unexplored topics in India. More than half of the older adults in India are migrants, and growing older at their destination place might cause a risk of lower cognitive health. To our knowledge, this is the first study to attempt to examine the association between a person’s age at migration and cognitive function and its variation with male and female older adults with national-level samples in India.

The study results indicate that females have a higher proportion of migration than males in India, and most migrants migrated through the rural-to-rural and rural-to-urban streams. In India, marriage is a prominent reason for migration, and after marriage, the female mainly migrates to her husband’s home [35, 36]. The migration from rural to rural occurs mainly due to the marriage region among females [36], while among males, due to new development activities in another rural area [37]. The persons migrated from rural to urban primarily due to work and employment in India [38]. Primarily in India, child migration occurs due to parental or family-related moves. In contrast, adult migration stems from factors such as marriage, education, and employment [25, 39], so most older migrants migrate early in life and grow at their migration place in India.

Our study shows that persons with migration status had lower cognitive scores than non-migrants, which aligns with previous research findings [9, 13, 21, 40]. Migration is a significant life event, and its associated changes, including socioeconomic status (SES), lifestyle, and environment, may significantly impact cognitive health in later life [14]. The studies reveal that due to migration, the chance of having depression symptoms may increase due to the separation from family, reduced social support, perceived discrimination, and a lack of sense of belonging to their destination place, which can affect cognitive function in old age [13, 41, 42]. In India, migrants show low social support compared to non-migrants [29], and studies show that low social support is significantly associated with lower cognition in later life [42, 43]. Understanding the life course perspective of migration’s effect on cognition is essential. However, social and physical exposures during and after the migration periods at various stages of life, such as early or childhood, mid-life, and old age, may have cumulative effects on health status in later life [44]. Our study shows that female migrants had lower cognitive scores than male migrants. Findings from studies support that females are more vulnerable to lower cognitive function in later life than male migrants. This is consistent with previous studies [45–47], which suggests that women are at a higher disadvantage in terms of having adverse structural, behavioural, and psychosocial characteristics across the lifespan that are related to poor late-life health outcomes.

Further, the age at migration shows a significant association with cognitive function in later life. In the unadjusted model, individuals who migrated at ages 0–14 and 60 years or above exhibited lower cognitive scores compared with non-migrants. However, after adjusting for socioeconomic and health-related characteristics, migrants who moved at ages 0–14, 15–44, and 45–59 years showed significantly lower cognitive scores than non-migrants, while those who migrated at age 60 years or above did not differ significantly from non-migrants. Previously, a few studies have shown that migration timing influences the health outcomes of migrants in older age [13, 47–49]. Migration at an early age provides more educational and occupational opportunities to accumulate financial resources. In contrast, the early age of migration largely shapes the duration of exposure to an environment and its associated resources or risks, such as separation, stress, social isolation, and limited access to health care, which may influence cognitive health later in life [11, 13, 14]. Migration during childhood may influence cognitive outcomes through prolonged exposure to both opportunities and risks at the destination. While early migration can provide greater access to education and occupational mobility, it may also involve early-life stress, social disruption, and prolonged exposure to disadvantaged environments or limited access to healthcare, which can have lasting consequences for cognitive health. In the Indian context, childhood migration often occurs as part of family economic migration from rural to urban areas or across regions in search of employment outcomes [26]. Such migration may involve disruptions in schooling, adjustment to new linguistic and social environments, and residence in economically disadvantaged urban settlements. Consequently, although early migration may offer potential opportunities, prolonged exposure to socioeconomic disadvantage and environmental stressors may contribute to poorer cognitive outcomes in later life.

Moreover, previous research has found that migrants who migrated in midlife are positively associated with health [18, 49]. Our results show that migrants in midlife are adversely associated with cognitive function in later life. Migration during midlife or later adulthood may be accompanied by reduced adaptive capacity, disruption of established social networks, and heightened psychosocial stress, potentially outweighing any economic or health advantages. Although previous studies have reported positive health associations among midlife migrants, our findings suggest that cognitive outcomes may be particularly sensitive to the timing of migration, reflecting cumulative stress exposure and limited opportunities to build cognitive reserve at later stages of life. Differences between the unadjusted and adjusted models indicate that part of the association between age at migration and late-life cognition is explained by socio-economic, demographic, and health factors accumulated across the life course. However, the persistence of lower cognitive scores for several age-at-migration groups after full adjustment suggests that migration and its timing may have independent effects on late-life cognition beyond these factors. These effects may reflect migration-related disruptions, such as long-term changes in social support, psychosocial stress, and challenges in adaptation, which are not fully captured by conventional covariates. The discrepancy between our findings and previous studies reporting positive health outcomes among midlife migrants may reflect differences in migration contexts. Much of the existing literature is based on high-income countries, where migration during midlife is often associated with occupational mobility and improved socioeconomic conditions [18, 49]. In contrast, midlife migration in India frequently occurs due to economic necessity, employment instability, or agrarian distress, and migrants are commonly engaged in informal or physically demanding occupations. These circumstances may increase psychosocial stress and reduce opportunities to accumulate cognitive reserve, potentially contributing to poorer cognitive outcomes in later life. In contrast, the absence of a significant association among those who migrated at age 60 years or above may reflect shorter exposure to migration-related stressors or possible health selection among older migrants.

Findings from sex-stratified analyses indicate that age at migration is differentially associated with cognitive function among male and female migrants in later life. Migration at an early age (0–14 years) and old age (45–59 and 60 + years) shows a negatively associated cognitive score in male and female migrants compared to their non-migrant reference. Subsequently, the result shows that migration at mid-life [15–44] was not significantly negatively associated with cognitive scores among male and female adults compared to non-migrant counterparts. The separation from one’s biological family of females may, in turn, cause more significant stress levels and low social support that could adversely affect cognitive function in later life [50, 51]. Further, various studies in India and China show that female migrants who migrated from rural-to-rural areas had a lower cognitive function in later life [21, 30, 52, 53]. The study on the cognitive gap between females and non-migrants in the U.S. shows that Mexican female migrants had lower cognition than non-migrant females [54]. Similar results were found in the study by Xu et al., 2020; the study revealed that female migrants had significantly lower cognitive function than males in China and India [46]. The males primarily migrated due to work and employment from rural to urban areas in India. Migrants’ health may be influenced by the poor quality of life in urban regions due to stress, acculturation, less access to healthcare, and more vulnerability to poor housing conditions [10, 13]. Two studies in India have examined the place of residence and cognitive relation among older adults, and the findings suggest that change in place of residence through life course is significantly associated, and the findings show that migrants with rural-to-rural and rural-to-urban residence were more vulnerable to poor cognitive health [9, 11] The finding from another study by Mohammad et al., 2022 revealed a significant female disadvantage in cognition and a stronger association of childhood health conditions with late-life cognitive functioning among women than men, where older women with fair health status in childhood had poorer cognitive performance in late-life compared to those with a good health status during childhood [55].

Furthermore, the cognition association with socioeconomic and health characteristics has been well exhibited in previous studies worldwide. The present study observed lower cognition scores among the oldest old adults, female, widowed, scheduled tribes, living with others, poor functional status, and poor self-rated health, compared to their reference category, which is also supported by similar findings from studies [24, 33, 56–60]. Subsequently, education, urban place of residence, wealth condition, and actively doing physical activity showed positive cognition among older adults in India, and these findings were supported by many studies conducted in India and other countries [4, 56, 60, 61].

The current study focused on the age of migration and its association with cognitive results in later life in India. Migrants have different characteristics from non-migrants, which play a vital role in health outcomes. This study examines not only cognitive differences with migration status but also how the age at migration is associated with cognition in late life. Before interpreting the findings of the research, some limitations should be considered. First, the absence of questions related to reasons for migration and the distance of migration, the absence of information on migration distance, limits our ability to examine how different types of migration may differentially influence late-life cognitive outcomes. Shorter-distance migration (for example, within nearby villages or districts) may allow greater continuity of social support, language, and cultural familiarity, whereas long-distance migration may involve more substantial social, cultural, and environmental disruption. The variable used in this study may not have adequately captured all aspects of people’s migration history, such as multiple migration histories. Second, the study is based on a cross-sectional design, which limits the paper’s findings regarding causal analysis, and findings do not imply clinically significant impairment at the individual level. The 60 + age group may result in wider confidence intervals and less precise estimates, whereas larger groups, such as those aged 0–14 years, yield more stable estimates. Although several comparisons showed statistically significant differences between migrants and non-migrants, the magnitude of these differences was relatively small. Given the large sample size, even small differences may achieve statistical significance, and it remains unclear whether these differences represent clinically meaningful variations in cognitive functioning. Therefore, the findings should be interpreted with caution, and future longitudinal studies incorporating clinical thresholds or cognitive impairment classifications may help better assess their real-world implications. Despite its limitations, this study used the most recent and national-level survey that has covered cognition function measures among adults in India. This data set will allow causal analysis when further survey wave data emerge.

## Conclusion

The present study found that older adult migrants in India show poorer late-life cognition than non-migrants, and that age at migration and gender differentially impact late-life cognitive outcomes. The insights gleaned from the study can significantly inform the formulation of policies and the execution of programs tailored to the needs of older individuals in terms of cognitive health. The findings highlight the complex associations between migration and cognitive function in later life and may have important implications for promoting healthy cognitive ageing among older adults in India.

## Data Availability

The study utilizes a secondary source of data that is freely available in the public domain through a request at the page https://www.iipsdata.ac.in/datacatalog_detail/5
